# Genetic Factors Influencing Coagulation Factor XIII B-Subunit Contribute to Risk of Ischemic Stroke

**DOI:** 10.1161/STROKEAHA.115.009387

**Published:** 2015-07-27

**Authors:** Ken B. Hanscombe, Matthew Traylor, Pirro G. Hysi, Stephen Bevan, Martin Dichgans, Peter M. Rothwell, Bradford B. Worrall, Sudha Seshadri, Cathie Sudlow, the METASTROKE Consortium, the Wellcome Trust Case Control Consortium 2, Frances M.K. Williams, Hugh S. Markus, Cathryn M. Lewis

**Affiliations:** From the Department of Medical & Molecular Genetics (K.B.H., C.M.L.), Department of Twin Research and Genetic Epidemiology (P.G.H., F.M.K.W.), and MRC Social, Genetic and Developmental Psychiatry Centre, Institute of Psychiatry, Psychology & Neuroscience (C.M.L.), King’s College London, London, UK; Department of Clinical Neurosciences, University of Cambridge, Cambridge, UK (M.T., S.B., H.S.M.); Institut für Schlaganfallund Demenzforschung, Klinikum der Universität München, Ludwig-Maximilians-Universität München, Feodor-Lynen-Straße 17, Munich, Germany (M.D.); Munich Cluster for Systems Neurology (SyNergy), Munich, Germany (M.D.); Stroke Prevention Research Unit, Nuffield Department of Clinical Neurosciences, University of Oxford (P.M.R.); Center for Public Health Genomics, and Cardiovascular Research Center, University of Virginia, Charlottesville, VA (B.B.W); Department of Biostatistics, Boston University School of Public Health, Boston, MA (B.B.W); Department of Neurology, Boston University School of Medicine, Boston, MA (S.S.); and Division of Clinical Neurosciences, University of Edinburgh, UK (C.S.).

**Keywords:** coagulation, genome-wide association study, stroke

## Abstract

**Background and Purpose—:**

Abnormal coagulation has been implicated in the pathogenesis of ischemic stroke, but how this association is mediated and whether it differs between ischemic stroke subtypes is unknown. We determined the shared genetic risk between 14 coagulation factors and ischemic stroke and its subtypes.

**Methods—:**

Using genome-wide association study results for 14 coagulation factors from the population-based TwinsUK sample (N≈2000 for each factor), meta-analysis results from the METASTROKE consortium ischemic stroke genome-wide association study (12 389 cases, 62 004 controls), and genotype data for 9520 individuals from the WTCCC2 ischemic stroke study (3548 cases, 5972 controls—the largest METASTROKE subsample), we explored shared genetic risk for coagulation and stroke. We performed three analyses: (1) a test for excess concordance (or discordance) in single nucleotide polymorphism effect direction across coagulation and stroke, (2) an estimation of the joint effect of multiple coagulation-associated single nucleotide polymorphisms in stroke, and (3) an evaluation of common genetic risk between coagulation and stroke.

**Results—:**

One coagulation factor, factor XIII subunit B (FXIIIB), showed consistent effects in the concordance analysis, the estimation of polygenic risk, and the validation with genotype data, with associations specific to the cardioembolic stroke subtype. Effect directions for FXIIIB-associated single nucleotide polymorphisms were significantly discordant with cardioembolic disease (smallest *P*=5.7×10^−04^); the joint effect of FXIIIB-associated single nucleotide polymorphisms was significantly predictive of ischemic stroke (smallest *P*=1.8×10^−04^) and the cardioembolic subtype (smallest *P*=1.7×10^−04^). We found substantial negative genetic covariation between FXIIIB and ischemic stroke (rG=−0.71, *P*=0.01) and the cardioembolic subtype (rG=−0.80, *P*=0.03).

**Conclusions—:**

Genetic markers associated with low FXIIIB levels increase risk of ischemic stroke cardioembolic subtype.

Altered coagulation predisposing to thrombosis has been suggested to play an important role in ischemic stroke, although the extent of this association, of which coagulation factors are the most important mediators, and whether association differs by ischemic stroke subtype, is unknown. One way to study thrombosis in the context of stroke is to consider commonalities in the underlying genetic liability to coagulation and ischemic stroke. Twin and family-based studies have demonstrated substantial genetic contribution to plasma concentrations of hemostatic proteins in the general population^1^ and thrombosis risk in the general population.^2^ Similarly, twin and family-based epidemiological studies, as well as more recent estimates of heritability from genome-wide association study (GWAS) arrays, suggest a substantial heritability for ischemic stroke.^3–5^

In a previous GWAS, we found that common genetic variants in the ABO gene were associated with ischemic stroke subtypes large-vessel disease (LVD) and cardioembolic (CE) stroke, but not with small-vessel disease (SVD).^6^ However, even though the GWAS approach surveys the entire genome for genetic associations, it typically queries one single nucleotide polymorphism (SNP) at a time for association with the disease or trait of interest. Evidence from GWASs suggests that common complex traits, such as coagulation and ischemic stroke, are polygenic, influenced by many SNPs each contributing a relatively small effect. Recently developed approaches test a polygenic model of common genetic liability by considering aggregate SNP effects.^7,8^ We used GWAS results for multiple different coagulation factors and hypothesized that by aggregating the evidence for association in each coagulation factor, we would be able to identify those coagulation factors that increase stroke risk and determine whether these differentially associate with stroke subtype.

## Methods

### Cohorts Studied

#### TwinsUK

The UK Adult Twin Registry (TwinsUK, http://www.twinsuk.ac.uk^9,10^) was the sampling frame for a European Union–funded multicentre study that aimed to identify common genetic risk for blood coagulation and ischemic stroke (EuroCLOT, http://cordis.europa.eu/result/rcn/52015_en.html6). Coagulation and fibrinolytic factors were selected on the basis of evidence for association with risk for atherothrombotic disorders, in a previous study estimating heritability of hemostatic factors.^1^ These hemostatic proteins are components of the coagulation pathway that is part of the defense mechanism against severe blood loss.^11^ The plasma protein assay protocols for the coagulation factors are described in detail elsewhere.^1,6^ Genotyping was performed in 3 batches on the Illumina Human Hap300 and Human Hap610-Quad arrays. Full details of genotyping and quality control are described elsewhere.^6^ The sample sizes for the 14 coagulation factors for which GWAS results were available are shown in Table. We used the coagulation GWAS results as a discovery sample.

**Table.**
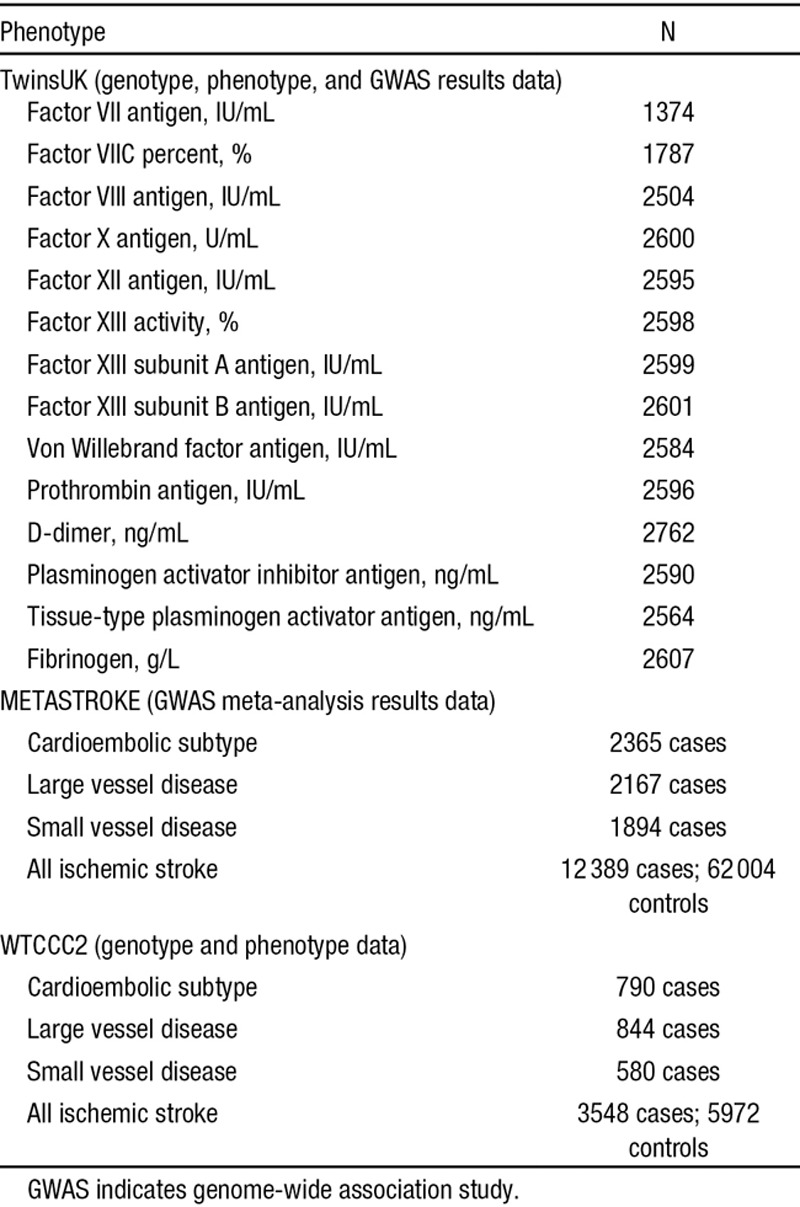
Study Sample Sizes

#### METASTROKE

METASTROKE is a 15-cohort meta-analysis of ischemic stroke GWASs.^12^ The GWASs included ischemic stroke patients of European ancestry (Europe, North America, and Australia) with ancestry-matched controls. Eleven studies used case–control methodology and were cross-sectional; the remaining 4 were prospective, population-based cohorts. Full details of the populations and analysis are described elsewhere.^12^ We used METASTROKE meta-analysis results as the target sample.

#### WTCCC2

The Wellcome Trust Case Control Consortium 2 (WTCCC2) ischemic stroke sample—the largest METASTROKE subsample—included a German (1174 cases and 797 controls) and a UK sample (2374 cases and 5175 controls).^13^ After combining the UK and German samples, we removed 16 population outliers. We used the WTCCC2 stroke sample genotypes as our validation sample.

In all METASTROKE studies, including the WTCCC2 subsample, stroke was defined as a typical clinical syndrome with radiological confirmation. Stroke subtyping was performed with the Trial of Org 10172 in Acute Stroke Treatment (TOAST) classification system.^14^ Brain imaging with computed tomography or magnetic resonance imaging was undertaken for >95% of cases in all the METASTROKE cohorts (sample sizes are included in Table).

### Study Design

#### SNP Selection

We used a thinned set of coagulation-associated risk SNPs for both the concordance and polygenic risk analyses because SNP correlation because of linkage disequilibrium may inflate test statistics from polygenic approaches.

##### Clumping

From the TwinsUK SNPs imputed to HapMap2, we first selected only SNPs common to both genotyping platforms used. For all SNPs with a minor allele frequency ≥0.05, we then used the clumping routine implemented in PLINK,^15^ which selects SNPs based on association *P* value and linkage disequilibrium, using standard parameter values (linkage disequilibrium threshold of *r*^2^ ≤0.25 in the HapMap CEU reference panel^16^; distance window of 300 kb). Clumping resulted in a subset of ≈70 000 near-independent SNPs for each coagulation factor. The ratio of estimated number of independent SNPs to observed (clumped) SNPs, the so-called effective ratio,^17^ was ≈0.93.

##### Inclusion Thresholds

We repeated each coagulation factor-stroke subtype analysis at multiple SNP-association *P* value inclusion thresholds. We ranked the clumped SNPs by their evidence for association in the TwinsUK discovery GWASs and created 7 overlapping SNP subsets for all SNPs significant at *P* ≤{0.01, 0.05, 0.1, 0.2, 0.3, 0.4, 0.5}, for each factor. These *P* values define increasingly liberal inclusion thresholds. Polygenic approaches show that more liberal inclusion thresholds benefit from true associations that exist all the way down a *P* value ranked list of genetic markers, far below the level usually considered as genome-wide significant.^7^

#### Multiple Testing

We estimated the number of independent coagulation variables using a Nyholt correction^18^ of the coagulation factor polygenic risk scores (PRSs). The number of independent tests from the 14 coagulation PRSs was estimated at 13.4, which highlights the low level of polygenic correlation among the coagulation factors themselves. We used a conservative Bonferroni-corrected probability value of *P*≤0.05/(13.4×4), where 4 is the number of stroke subtypes, to control for multiple testing in the discovery and target samples using GWAS summary statistics; we used *P*≤0.05 for focused follow-up on genotype data in the validation subsample.

### Statistical Analysis

We used 3 approaches to test for shared genetic influence on coagulation traits and ischemic stroke and its subtypes, as follows:

#### Concordance of Genetic Effects

We first tested for greater than expected concordance (or discordance) in direction of effects (β-estimate, odds ratio) between each coagulation factor–stroke subtype pair. At each *P* value inclusion threshold, we used an Exact Binomial Test to assess whether there was a greater than chance agreement in effect direction. This approach simply counts the number of SNPs acting in the same direction, regardless of magnitude of effect and tests the expectation under the null hypothesis of 50% agreement.

#### Polygenic Risk Scores

Next we assessed shared genetic risk (pleiotropy) between each coagulation factor and each stroke subtype by summing over all SNP effects in the target sample and weighting by evidence for association in the coagulation discovery sample. For a given set of SNPs, an individual’s PRS is the sum of risk alleles they have weighted by allele effect size in the discovery trait. It represents their genetic risk for the discovery trait. The method described below does not explicitly calculate the coagulation PRS for each individual but rather estimates the association between such a score and the target trait.

#### Summary Statistics

With summary data, the joint effect of the SNPs in each subset was estimated by a weighted mean of individual SNP effects, that is, summing the SNP effects in the stroke target sample weighted by their effects in the coagulation discovery sample. In a regression model predicting the target sample outcome, *y* = *c* + *a*PRS + *e*, the PRS coefficient can be estimated by


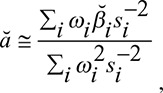


where 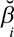 are the discovery sample SNP effects, *ω*_*i*_ are the target sample SNP effects, and *s*_*i*_ are the target sample standard errors for the *i*th SNP. Without individual-level genotype data, this method does not explicitly estimate a PRS—only its effect. The joint SNP effect estimate inherits covariate adjustment from covariates included in the contributing GWASs, for example, population structure axes.^19^ The approach (described in detail elsewhere^20^) is implemented in the package gtx (gtx: Genetics ToolboX, http://cran.r-project.org/web/packages/gtx)^21^ in the language and statistical computing environment R (http://www.R-project.org/).^22^

#### Genetic Covariance

Finally, using genotype-level data for the TwinsUK sample and the UK and German subsamples of the WTCCC2, we validated findings from the concordance and polygenic risk analyses by estimating the phenotypic covariation between the 2 traits (ie, pleiotropy—rG_SNP_), as well as genetic contribution to phenotypic variance within trait (ie, univariate SNP heritability—*h*2_SNP_). These estimates are based on distant relatedness calculated from common SNPs. Estimation of SNP heritability and pleiotropy between complex traits using SNP-derived genomic relationships and restricted maximum likelihood is implemented in the program Genome-wide Complex Trait Analysis.^8,23^ Specifically, we used the genetic covariance estimation with genotype data to validate findings from the concordance and polygenic risk analyses with GWAS summary results.

## Results

### Concordance of Genetic Effects

**Figure 1.**
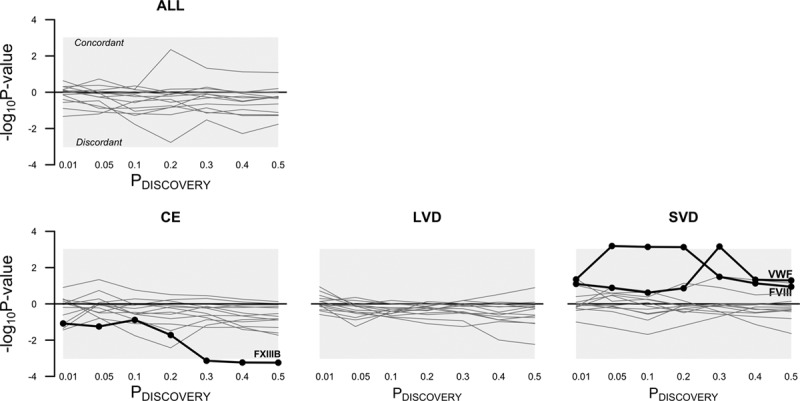
Common direction of effects in coagulation and stroke. *P* value inclusion thresholds define SNP subsets based on significance in the coagulation factor genome-wide association studies (GWASs). For example, at *P*≤0.5 (the most liberal inclusion threshold), half the SNPs from each GWAS discovery study were used to test for concordance of effect between the coagulation discovery samples and the stroke target samples. The *y*-axes show evidence of excess similarity (or dissimilarity) in direction of effect (negative log_10_ *P* values from an Exact Binomial Test). Each line in a plot represents strength of evidence that a particular coagulation factor’s genetic risk in aggregate acts in the same (or opposite) direction to the genetic risk for stroke. Factors with significant similarity (or dissimilarity) of effect are highlighted—these traits survive multiple test correction (*P*≤0.5/(13.4×4)) and lie outside the grey-shaded area. ALL indicates all ischemic stroke; CE, cardioembolic subtype; FVIII, factor VIII; LVD, large-vessel disease subtype; –log_10_*P* value, *P* value from the binomial test for concordance; *P*_DISCOVERY_, *P* value inclusion threshold defining SNP subsets; SVD, small-vessel disease subtype; and VWF, Von Willebrand factor.

Three coagulation factors showed excess concordance (or discordance) of effect direction with stroke: SNPs significant at *P*≤0.3 in factor XIII subunit B (FXIIIB) were significantly discordant with the CE subtype (most significant *P*=5.7×10^−04^); SNPs significant at *P*≤0.05 through *P*≤0.2 in factor VIII (FVIII) were significantly concordant with the SVD subtype (most significant binomial *P*=6.5×10^−04^), but not at more liberal inclusion thresholds; SNPs significant at *P*≤0.3 only in Von Willebrand factor were significantly concordant with the SVD subtype. Figure 1 summarizes the evidence for excess concordance (or discordance) of effect between the 14 coagulation factors and ischemic stroke and its 3 subtypes.

### Polygenic Risk Scores

**Figure 2.**
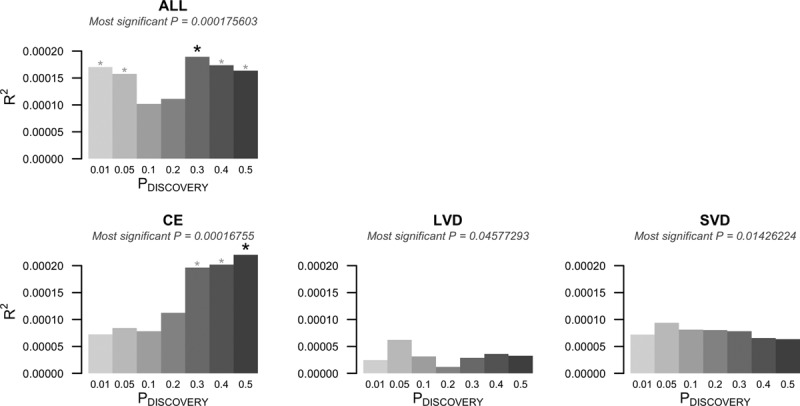
Common polygenic risk for factor XIII subunit B (FXIIIB) and stroke. The figure shows the polygenic risk prediction of FXIIIB-associated risk for all ischemic stroke and the ischemic stroke subtypes. ALL indicates all ischemic stroke; CE, cardioembolic subtype; GWAS, genome-wide association studies; *P*_DISCOVERY_, *P* value inclusion threshold defining SNP subsets from coagulation factor GWASs; *R*^2^, variance explained by the polygenic risk score (PRS; pseudo *R*^2^ from a logistic regression); and SNP, single nucleotide polymorphism. *Inclusion thresholds that significantly predict stroke status (bold asterisk shows most significant threshold).

All ischemic stroke and the CE subtype, but not the LVD or SVD subtypes, were significantly associated with coagulation polygenic risk. For all ischemic stroke, PRSs from 3 coagulation factors were significant: FVIIC% at inclusion thresholds *P*≤0.2 and 0.4 (most significant *P*=4.9×10^−04^); FX at inclusion threshold greater than *P*≤0.4 (most significant *P*=1.5×10^−04^); and FXIIIB for inclusion thresholds greater than *P*≤0.01 (most significant *P*=1.8×10^−04^). For the CE subtype, FXIIIB was significantly predictive at all inclusion thresholds greater than *P*≤0.2 (most significant *P*=1.7×10^−04^), although the proportion of variance explained (*R*^2^) was modest (<1%) at all inclusion thresholds. FXIIIB was the one coagulation factor that showed consistent effects in both the concordance analyses and the PRS analyses. Figure 2 shows the increasing explanatory power of FXIIIB for all ischemic stroke and CE, as we increased the number of FXIIIB-associated risk variants included in the stroke prediction model.

In the target sample analyses, we found that genetic variation associated with lower FXIIIB levels was consistently predictive of higher stroke risk: genetic risk was significantly discordant for FXIIIB and stroke, and a negative association between polygenic risk based on FXIIIB indicates that controls had more FXIIIB-associated SNPs than stroke cases.

### Genetic Covariation

Finally, to validate the association with FXIIIB using an alternative approach, we directly estimated from genotype data the genetic covariation between stroke and FXIIIB. We found evidence of a significantly high level of shared genetic risk for FXIIIB–all ischemic stroke (rG_SNP_=−0.71, SE=0.81, *P*=0.01, N=10,668; *h*2_SNP_ FXIIIB =0.13, SE=0.28; *h*2_SNP_ ischemic stroke =0.45, SE=0.04). Similarly, bivariate GREML analysis for FXIIIB–CE suggested a significantly high degree of common genetic risk (rG_SNP_=−0.80, SE=0.90, *P*=0.03, N=7917; *h*2_SNP_ FXIIIB =0.13, SE=0.28; *h*2_SNP_ CE =0.34, SE=0.05). The negative genetic covariance between FXIIIB and both all ischemic stroke and the CE subtype indicates that genetic variation predisposing to low levels of FXIIIB is associated with increased risk for stroke. A negative genetic correlation is consistent with our results above: FXIIIB had discordant SNP effects with CE stroke and the FXIIIB-associated PRS for stroke was lower in cases.

The large standard errors associated with these estimates are typical for small sample sizes in bivariate GREML analyses. From the combined TwinsUK-WTCCC2 sample, we excluded one individual from each pair with an estimated relatedness above 10%. We chose this cut-off (which removes up to first cousin relationships) because of the coagulation sample size: for example, of the 10 668 individuals in the FXIIIB-ischemic stroke analysis, 1940 were TwinsUK samples. Using the default Genome-wide Complex Trait Analysis relatedness cut-off of 2.5%, we found similarly high point estimates with much larger standard errors (FXIIIB-ischemic stroke: rG_SNP_=−0.85, SE=2.69, *P*=0.06), suggesting that a larger sample would yield similar pleiotropy estimates with narrower confidence intervals.

## Discussion

Here, we used statistical genetic approaches to evaluate the genetic influence of coagulation traits on ischemic stroke and its subtypes. Our results indicate that the aggregate effect of risk SNPs for the plasma protein FXIIIB has a small, but significant effect on risk for ischemic stroke, specifically for the CE subtype, but not LVD or SVD stroke. Genetic factors that increased levels of FXIIIB were associated with decreased ischemic stroke risk. This result was identified using 3, but related, statistical analysis approaches.

A common genetic contribution to higher levels of FXIIIB and lower risk of ischemic stroke might seem counterintuitive. Two A subunits (FXIIIA) and 2 B subunits (FXIIIB) make up the FXIII tetramer whose main function is to strengthen and protect the fibrin clot against degradation during clot formation. Individuals with FXIII deficiency manifest a severe susceptibility to hemorrhage (or bleeding diathesis).^24^ However, as well as its clot stabilizing or prothrombotic effects, FXIII also has an antithrombotic effect by inhibiting platelet aggregation.^25^ That said, it is not clear what effect high plasma levels of free-floating FXIIIB (measured by the assay) would have on levels of the FXIII tetramer and how a change in levels of FXIII would affect the balance of its pro- and antithrombotic effects. At a 2.8-year poststroke follow up for mortality, FXIIIB and FXIIIA have been found to be present at significantly higher levels in survivors compared with those that had died.^26^ One particular polymorphism, FXIIIVal34Leu, has been reported to provide a protective effect against venous thromboembolism and myocardial infarction in Caucasians (in the absence of insulin resistance).^27,28^ The same polymorphism conveys no protective effect in South Asians, known to have higher levels of insulin resistance. FXIIIB was in fact found to be higher among South Asian ischemic stroke cases even after accounting for insulin resistance.^29^ However, as polygenic risk scoring approaches by design capture net genetic effects, the risk score based on FXIIIB-associated SNPs is the sum of many SNPs (of which the base change at the FXIIIVal34Leu polymorphism is just one), potentially acting both to increase and decrease levels of FXIIIB. The biological role of FXIIIB is complex, and further research is required to understand the specific mechanism underlying the observed protective effect.

In contrast, we found no association with LVD. Thromboembolism is believed to be the primary pathophysiological mechanism in LVD as in CE, but the underlying thrombotic mechanisms may differ in LVD. This is supported by clinical trial data, suggesting antiplatelet agents seem to be the most effective secondary prevention approach in LVD,^30^ although it is worth noting that the superiority of antiplatelets over anticoagulants for ischemic stroke of non-CE origin is not unequivocal.^31^ In contrast, anticoagulants have consistently been shown to be more effective than antiplatelets in CE stroke. There was also no association with SVD, and this would be consistent with a lack of pathophysiological evidence linking thrombosis with this stroke subtype.^32^

This study does have limitations. First, although our stroke sample was relatively large, numbers of cases in the subtype analyses were necessarily smaller. Similarly, sample sizes for the coagulation factor GWASs were modest. Second, we benefitted from aggregating evidence across many SNPs, including those that did not achieve genome-wide significance in earlier genetic studies of coagulation and stroke, but this provides no resolution at individual SNPs, and we were consequently unable to comment on specific FXIIIB polymorphisms previously reported to be associated with thrombotic disease (stroke). Third, our final technique that tested genetic covariation used a subset of the METASTROKE sample and so was not a true replication in an independent data set. However, because the genetic covariation technique used SNP-level information for each individual in both the coagulation and stroke studies (as opposed to summary statistics from the overall GWASs), we included it as a validation of polygenic risk estimation using only GWAS meta-analysis results from the stroke studies.

A recent review of the role of FXIII in the risk for thrombotic diseases found only 4 studies that investigated FXIII levels and ischemic stroke.^25^ It concluded that no clear picture emerged and called for well-designed studies with clear differentiation of stroke subtype to clarify the problem. By aggregating evidence across the entire genome, our study found a link between the genetic influence on FXIIIB levels and the CE subtype, narrowing the focus to a specific subtype and highlighting a direction for further investigation into the mechanism behind the role of FXIIIB in aberrant coagulation and the pathogenesis of ischemic stroke.

## Sources of Funding

Funding for collection, genotyping, and analysis of stroke samples was provided by Wellcome Trust Case Control Consortium-2. We acknowledge the use of the British 1958 Birth Cohort DNA collection and UK National Blood Service controls. The research was funded by the National Institute for Health Research Biomedical Research Centre based at Guy’s and St Thomas’ National Health Service Foundation Trust and King’s College London, and the Biomedical Research Centre for Mental Health at South London and Maudsley NHS Foundation Trust and King’s College London. The views expressed are those of the author(s) and not necessarily those of the National Health Service, the National Institute for Health Research, or the Department of Health. H.S. Markus is supported by a National Institute for Health Research Senior Investigator award. H.S. Markus and S. Bevan are supported by the National Institute for Health Research Cambridge University Hospitals Comprehensive Biomedical Research Centre. M. Traylor is supported by a project grant from the Stroke Association (TSA 2013/01). B.B. Worrall is supported by a grant from the National Institutes of Health (NIH).

## Disclosures

Dr Worrall is an associate editor for Neurology. The other authors report no conflicts.

## References

[1] de Lange M, Snieder H, Ariëns RA, Spector TD, Grant PJ. (2001). The genetics of haemostasis: a twin study.. Lancet.

[2] Souto JC, Almasy L, Borrell M, Blanco-Vaca F, Mateo J, Soria JM (2000). Genetic susceptibility to thrombosis and its relationship to physiological risk factors: the GAIT study. Genetic analysis of idiopathic thrombophilia.. Am J Hum Genet.

[3] Brass LM, Isaacsohn JL, Merikangas KR, Robinette CD. (1992). A study of twins and stroke.. Stroke.

[4] Bak S, Gaist D, Sindrup SH, Skytthe A, Christensen K. (2002). Genetic liability in stroke: a long-term follow-up study of Danish twins.. Stroke.

[5] Jerrard-Dunne P, Cloud G, Hassan A, Markus HS. (2003). Evaluating the genetic component of ischemic stroke subtypes: a family history study.. Stroke.

[6] Williams FM, Carter AM, Hysi PG, Surdulescu G, Hodgkiss D, Soranzo N, EuroCLOT Investigators; Wellcome Trust Case Control Consortium 2; MOnica Risk, Genetics, Archiving and Monograph; MetaStroke; International Stroke Genetics Consortium (2013). Ischemic stroke is associated with the ABO locus: the EuroCLOT study.. Ann Neurol.

[7] Purcell SM, Wray NR, Stone JL, Visscher PM, O’Donovan MC, Sullivan PF, International Schizophrenia Consortium, International Schizophrenia Consortium (2009). Common polygenic variation contributes to risk of schizophrenia and bipolar disorder.. Nature.

[8] Yang J, Lee SH, Goddard ME, Visscher PM. (2011). GCTA: a tool for genome-wide complex trait analysis.. Am J Hum Genet.

[9] Moayyeri A, Hammond CJ, Hart DJ, Spector TD. (2013). The UK Adult Twin Registry (TwinsUK Resource).. Twin Res Hum Genet.

[10] Spector TD, Williams FM. (2006). The UK Adult Twin Registry (TwinsUK).. Twin Res Hum Genet.

[11] Dahlbäck B. (2000). Blood coagulation.. Lancet.

[12] Traylor M, Farrall M, Holliday EG, Sudlow C, Hopewell JC, Cheng YC, Australian Stroke Genetics Collaborative, Wellcome Trust Case Control Consortium 2 (WTCCC2); International Stroke Genetics Consortium (2012). Genetic risk factors for ischaemic stroke and its subtypes (the METASTROKE collaboration): a meta-analysis of genome-wide association studies.. Lancet Neurol.

[13] Bellenguez C, Bevan S, Gschwendtner A, Spencer CCA, ISGC, WTCCC, ISGC, WTCCC (2012). Genome-wide association study identifies a variant in HDAC9 associated with large vessel ischemic stroke.. Nat Genet.

[14] Adams HP, Bendixen BH, Kappelle LJ, Biller J, Love BB, Gordon DL (1993). Classification of subtype of acute ischemic stroke. Definitions for use in a multicenter clinical trial. TOAST. Trial of Org 10172 in Acute Stroke Treatment.. Stroke.

[15] Purcell S, Neale B, Todd-Brown K, Thomas L, Ferreira MA, Bender D (2007). PLINK: a tool set for whole-genome association and population-based linkage analyses.. Am J Hum Genet.

[16] The International HapMap Consortium. (2005). A haplotype map of the human genome. Nature.

[17] Li MX, Yeung JM, Cherny SS, Sham PC. (2012). Evaluating the effective numbers of independent tests and significant p-value thresholds in commercial genotyping arrays and public imputation reference datasets.. Hum Genet.

[18] Nyholt DR. (2004). A simple correction for multiple testing for single-nucleotide polymorphisms in linkage disequilibrium with each other.. Am J Hum Genet.

[19] Ehret GB, Munroe PB, Rice KM, Bochud M, Johnson AD, Chasman DI, International Consortium for Blood Pressure Genome-Wide Association Studies; CARDIoGRAM consortium; CKDGen Consortium; KidneyGen Consortium; EchoGen consortium; CHARGE-HF consortium (2011). Genetic variants in novel pathways influence blood pressure and cardiovascular disease risk.. Nature.

[20] Dastani Z, Hivert MF, Timpson N, Perry JR, Yuan X, Scott RA, DIAGRAM+ Consortium; MAGIC Consortium; GLGC Investigators; MuTHER Consortium; DIAGRAM Consortium; GIANT Consortium; Global B Pgen Consortium; Procardis Consortium; MAGIC investigators; GLGC Consortium (2012). Novel loci for adiponectin levels and their influence on type 2 diabetes and metabolic traits: a multi-ethnic meta-analysis of 45,891 individuals.. PLoS Genet.

[21] Johnson T. (2012). Efficient calculation for multi-SNP genetic risk scores. American Society of Human Genetics Annual Meeting.

[22] R Core Team (2015). R: A language and environment for statistical computing.

[23] Lee SH, Yang J, Goddard ME, Visscher PM, Wray NR. (2012). Estimation of pleiotropy between complex diseases using single-nucleotide polymorphism-derived genomic relationships and restricted maximum likelihood.. Bioinformatics.

[24] Hashiguchi T, Saito M, Morishita E, Matsuda T, Ichinose A. (1993). Two genetic defects in a patient with complete deficiency of the b-subunit for coagulation factor XIII.. Blood.

[25] Bagoly Z, Koncz Z, Hársfalvi J, Muszbek L. (2012). Factor XIII, clot structure, thrombosis.. Thromb Res.

[26] Kohler HP, Ariëns RA, Catto AJ, Carter AM, Miller GJ, Cooper JA (2002). Factor XIII A-subunit concentration predicts outcome in stroke subjects and vascular outcome in healthy, middle-aged men.. Br J Haematol.

[27] Catto AJ, Kohler HP, Coore J, Mansfield MW, Stickland MH, Grant PJ. (1999). Association of a common polymorphism in the factor XIII gene with venous thrombosis.. Blood.

[28] Kohler HP, Stickland MH, Ossei-Gerning N, Carter A, Mikkola H, Grant PJ. (1998). Association of a common polymorphism in the factor XIII gene with myocardial infarction.. Thromb Haemost.

[29] Kain K, Young J, Bavington J, Bamford J, Catto AJ. (2005). Coagulation factor XIII B subunit antigen, FXIIIVal34Leu genotype and ischaemic stroke in South Asians.. Thromb Haemost.

[30] Iso H, Hennekens CH, Stampfer MJ, Rexrode KM, Colditz GA, Speizer FE (1999). Prospective study of aspirin use and risk of stroke in women.. Stroke.

[31] Mohr JP, Thompson JL, Lazar RM, Levin B, Sacco RL, Furie KL, Warfarin-Aspirin Recurrent Stroke Study Group (2001). A comparison of warfarin and aspirin for the prevention of recurrent ischemic stroke.. N Engl J Med.

[32] Hassan A, Hunt BJ, O’Sullivan M, Parmar K, Bamford JM, Briley D (2003). Markers of endothelial dysfunction in lacunar infarction and ischaemic leukoaraiosis.. Brain.

